# Systematic review: Efficacy of off-label antipsychotic use in children and adolescents with obsessive-compulsive, mood dysregulation, depression, anxiety, and sleep-related disorders

**DOI:** 10.1007/s00787-026-03049-9

**Published:** 2026-05-18

**Authors:** Akseli K. Holopainen, Pyry A. Vainiotalo, Bianca P. Arrhenius, Ida Ringbom, Mirja Laurila, David Gyllenberg

**Affiliations:** 1 Faculty of Medicine, University of Helsinki, Helsinki, Finland; 2 Finnish Medical Society Duodecim, Helsinki, Finland; 3 Department of General Practice and Primary Health Care, University of Helsinki, Helsinki, Finland; 4 Department of Adolescent Psychiatry, Helsinki University Hospital, Helsinki, Finland; 5 Department of Child Psychiatry, University of Turku, Turku, Finland

**Keywords:** Antipsychotics, Efficacy, Off-label, OCD, Mood disorder, Adolescent

## Abstract

Antipsychotics (APs) are not officially approved for the treatment of depressive, anxiety, obsessive-compulsive, emotional dysregulation, or sleep disorders among children and adolescents. Despite this, the treatment of these common and comorbid disorders is likely to partially explain the increased AP use. We assessed all studies reporting changes in symptoms or functioning during antipsychotic treatment for these diagnoses among patients < 18 years old. We systematically searched MEDLINE, EBM reviews and PsycINFO databases for all studies published by October 30, 2025, reporting on the effectiveness of APs for obsessive-compulsive, mood dysregulation, depression, anxiety, and sleep-related disorders among patients < 18 years. We evaluated evidence using the Grading of Recommendations Assessment, Development and Evaluation approach (GRADE). Of the 2237 identified studies, 119 were eligible for full-text review, and 13 were included in the final review. One study compared two antipsychotics, while the rest were uncontrolled open-label studies, case series, or case reports. No randomized controlled trials nor studies on sleep or anxiety disorders were identified. While there were reported changes in standardized mean scores between baseline and endpoint, the level of evidence in obsessive-compulsive, mood dysregulation, and depression related disorders was very low. The identified very low level of evidence stands in stark contrast to clinical practice, where APs are increasingly prescribed. Future methodologically robust studies are needed to demonstrate efficacy. Given the side effects of APs, physicians should carefully consider the benefits and harms when prescribing them for off-label indications.

**Supplementary Information:**

The online version contains supplementary material available at https://doi.org/10.1007/s00787-026-03049-9.

## Introduction

The use of antipsychotics (APs) among children and adolescents is prevalent and has been on the rise for the past three decades in the Western world. For example, the proportion of 15–19-year-old females using APs doubled from 3.9 to 7.9 per 1,000 in Germany between 2011 and 2020 [1–3]. In Nordic countries, between the years 2008 and 2017 prevalence increased among children and adolescents in Norway, Sweden, and Finland by 45%, 58% and 79%, respectively [4]. However, as shown in Supplementary table 1a (See Online resource 1), only schizophrenia, bipolar disorder, and persistent aggression associated with conduct disorders have been approved by the European Medicines Agency (EMA) as indications for AP use in children and adolescents [5]. These conditions are relatively rare. Therefore, a large proportion of AP use in child and adolescent populations is off-label, as is the case in conditions such as obsessive-compulsive (OCD), mood dysregulation, depression, anxiety, and sleep-related disorders [3]. This is increasingly evident with the rising prevalence of anxious and depressive symptoms in child and adolescent populations [6]. AP use among children and adolescents is associated with several serious metabolic and other adverse effects.[7; 8] Hence, clinicians need up-to-date knowledge on the efficacy of APs for off-label indications in order to make informed decisions on prescribing.

Previous systematic reviews on the efficacy of APs for unstable mood, depression, and anxiety-related disorders have reported a lack of evidence for efficacy in children and adolescents.[7; 9] However, previous systematic reviews have either studied only some of our included disorders [7], searched only for randomized controlled trials [9], or focused more on on-label indications [10]. Clinical guidelines are also crucial for gaining an overview of both current evidence and clinical practices. Noteworthy is that APs are seldom mentioned in clinical guidelines for the treatment of unstable mood, depression, and anxiety-related disorders in patients younger than 18 years. For example, the latest American Academy of Child and Adolescent Psychiatry guideline for depressive disorders from 2023 and anxiety disorders from 2020 do not include a single mention of APs.[11; 12] At the same time, prevalence studies have reported that up to 6.8% of adolescent inpatients with major depressive disorder have been prescribed antipsychotics in the United States [13]. When guidelines do mention APs, they rarely recommend use among adolescents and consistently urge for close monitoring and short-term use if they are prescribed. For example, the British NICE guideline for the identification and management of depression in children and young people from 2019 recommends AP use only in psychotic depression or as an augmentation therapy in treatment-resistant cases [14]. Similarly, the American Academy of Child and Adolescent Psychiatry guideline for OCD from 2012 recommends careful consideration before prescribing APs for children. It advises use only as a second-line augmentation option in treatment-resistant cases, particularly in those patients with a comorbid tic disorder [15]. 

Since previous systematic reviews have failed to confirm the efficacy of off-label use in children and adolescents, it is worthwhile to consider the strength of the evidence for adult populations. For example, a network meta-analysis of the treatment of unipolar depression reported that both aripiprazole and risperidone show superior response and remission rates compared to placebo when the APs were augmented to selective serotonin reuptake inhibitors (SSRIs) [16]. Similarly, an umbrella review from 2024 reported quetiapine being superior to placebo in the treatment of generalized anxiety disorder with moderate effect sizes in previous systematic reviews [17]. These findings are in line with the fact that the range of EMA-approved indications of APs is broader for adults than for children and adolescents. For instance, quetiapine has been approved for the treatment of treatment-resistant depression in adults, but not in children and adolescents [18]. 

Though AP use is on the rise, neither previous systematic reviews nor clinical guidelines provide a broad overview with standardized evidence assessments of their efficacy in children and adolescent populations. Therefore, a comprehensive overview of AP use in depression, OCD, unstable mood, anxiety, and sleep disorders is lacking. We aimed to fill this gap through a rigorous review of all studies conducted on these indications, regardless of study type, and assess their level of evidence.

## Methods

This systematic review was conducted in accordance with the Preferred Reporting Items for Systematic reviews and Meta-Analyses (PRISMA) statement. The review was registered in the International Prospective Register of Systematic Reviews (PROSPERO) on the 1st of July 2024 (registration number CRD42024559953). All amendments made to the a priori study protocol are reported online. These include specifying OCD as a part of the anxiety spectrum and therefore including it in the search terms, broadening search terms to identify all studies pertaining to mood disorders, changing our formula for calculating standardized mean change to match formula for Cohen’s *d* instead of Hedge’s *g* since there is negligible difference in accuracy of results and Cohen’s *d* is more widely recognized, as well as updating general information to the new PROSPERO record format.

### Search strategy

Our systematic search was conducted using OVID interface and various databases from their inception to October 2025 (Ovid MEDLINE ALL 1946-, EBM Reviews, APA PsycINFO 1806-, APA PsycArticles). Reverse snowballing was used to find additional relevant studies. The initial search took place on July 2, 2024. The search strategy and search terms were reviewed and refined by the University of Helsinki’s library information specialist. The search strategy included terms describing the indications for use and the study population, as well as the names of specific APs. An updated search to identify any additional recent publications was conducted on October 30, 2025. See Online Resource 2 for the complete search strategy.

### Study selection

Articles were considered eligible for inclusion if: (1) they examined the efficacy of off-label use of antipsychotics, (2) the study population included subjects under 18 years of age (3) study results for subjects under 18 years of age could be extracted separately with minimal or no risk of including older individual(s) (see Table 1. footnotes) (4) the research subjects were diagnosed with OCD, unstable mood, depression, anxiety and/or sleep related disorders, (5) the articles were written in English. Articles were excluded if their subjects did not include adolescents or if they were diagnosed with bipolar disorder, schizophrenia, psychotic episodes, or intellectual disability. Further, we excluded reviews that contained no primary data. See Online Resource 3 for the full list of articles that were excluded after full-text review and reasons for exclusions. All other published articles that met the inclusion criteria were included.

**Fig. 1:**
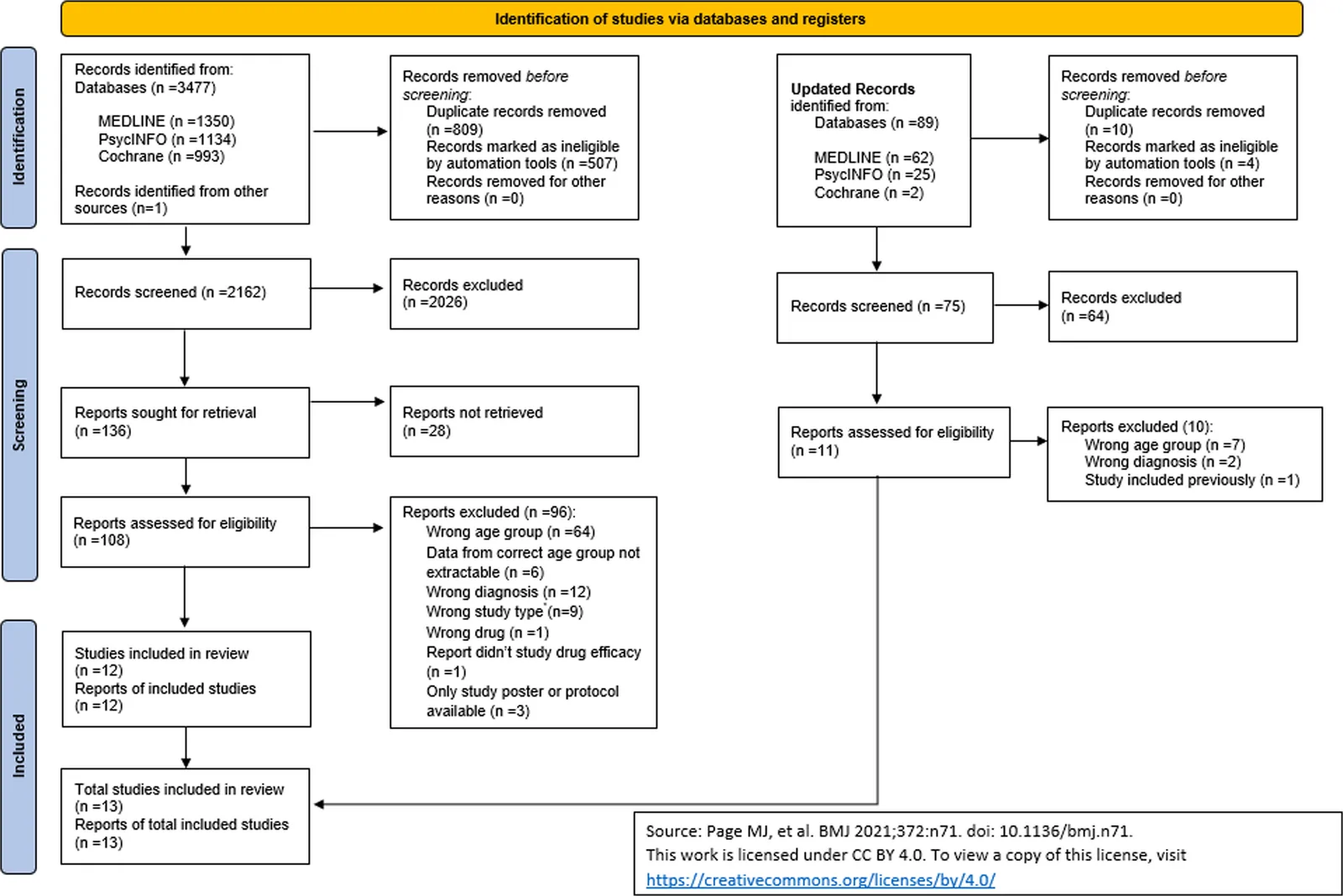
PRISMA flow chart. *Wrong study type includes articles with no original data, e.g., reviews

The screening for eligibility was conducted by two independent researchers (A.H. and P.V.), and any conflicts were resolved by a senior researcher (D.G.). The whole study selection procedure is described in the PRISMA flow chart (Fig. 1).

### Data extraction

After screening, all relevant data were extracted independently by two researchers (A.H. and P.V.) and placed in an Excel spreadsheet. The data included title, author, year, country, study design, AP used in the study, diagnosis, main outcome variables, study duration, sample size, mean age, AP dose, concomitant medications, and key findings associated with APs and their efficacy.

### Quality assessment

The quality of the included studies was assessed independently by two researchers (A.H. and P.V.) using the Grading of Recommendations Assessment, Development and Evaluation (GRADE) approach. Any disagreements were resolved by a senior researcher (D.G./B.A.) Factors lowering the quality of evidence were: (1) limitations in study design or execution (risk of bias), (2) inconsistency of results, (3) indirectness of evidence, (4) imprecision, and (5) publication bias. Factors that increased the quality of evidence were: (1) a large magnitude of effect, (2) all plausible confounders would reduce the demonstrated effect or increase the effect if no effect was observed, and (3) a dose-response gradient. Every article was categorized with a quality rating with values of ‘very low’, ‘low’, ‘moderate’, or ‘high’ certainty of evidence [19]. If an observational study has no strengths that increase the quality of evidence, it provides ‘low certainty of evidence’, whereas a randomized controlled trial with no important limitations offers ‘high certainty of evidence’. As stated in the GRADE handbook, the quality of evidence reflects the extent to which we are confident that an estimate of the effect is correct [19]. 

### Synthesis of results

After data extraction and quality assessment, we summarized the results. As most symptoms and global functioning instruments reported in the original work were assessed on different scales, for descriptive purposes, we standardized the outcome measures before and after treatment to make them as comparable as possible. We planned to conduct meta-analyses if the search results met our a priori criteria, which included at least three studies with comparable outcome variables. These studies had to use the same clinical scales, have similar treatment durations and doses for all patients (or data for similar patients could be extracted), and provide sufficient data for data pooling. We aimed to assess possible publication bias by creating funnel plots if more than ten of the included studies reported effect sizes and standard errors of similar outcome variables.

It is worth noting that some of the included studies used diagnoses that have been subsumed under other diagnoses in modern diagnostic classifications, or their names differ across classifications. Examples include the reclassification of severe mood dysregulation under disruptive mood dysregulation disorder, and emotionally unstable personality disorder being an alternative name for borderline personality disorder. All diagnoses have been reported according to the modern Diagnostic and Statistical Manual of Mental Disorders, 5th edition (DSM-5) classifications in this study.

We set out to organize information in a clear manner for readability purposes. Therefore, all included studies were divided under obsessive compulsive disorder, mood dysregulation and borderline disorders, or depression when presenting study characteristics and results.

## Results

A total of 3566 records were identified in the database search. One additional article was found using the reverse snowballing technique [20]. After duplicates were removed, 2237 studies remained for screening. Of these, 119 were eligible for full-text review. Finally, 13 studies were included in the review (Fig. 1, PRISMA flow chart).

We identified seven case series, five open-label trials (one of which included two treatment groups), and one case report. No randomized controlled trials meeting our inclusion criteria were identified. As shown in Table 1, five of the 13 included studies were on APs and OCD, five studied APs’ effect in the treatment of unstable mood regulation or borderline personality-related disorders, and two examined the effect of APs on depression. Additionally, one case series had patients with major depressive disorder and mood disorders. No studies on APs and anxiety or sleep disorders were identified. The total numbers of patients per condition were as follows: OCD (*n* = 137), mood dysregulation (*n* = 91), and depression (*n* = 13).

**Table 1:** Study characteristics

Study author	Year	Country	Study design	Study population	Sociodemo-graphic characte-ristics	Intervention (AT / MT)	Daily dosage, mean (SD)	Number of patients at baseline / at endpoint	Patient age in years, mean (SD)	Males, %	Primary outcome (generic)
**Obsessive-compulsive disorder**											
Ercan et al.	2015	Turkey	Open-label uncontrolled study	Treatment- resistant OCD	Socio-economic level of family: Good: 18.8% Middle: 37.5% Poor: 43.8%	aripiprazole (MT)	4.75 mg (NA)	16/16	10.9 (2.9)	43.8	Change in OCD symptoms
Masi et al.	2013	USA	Cohort study with two treatment groups	Treatment- resistant tic-related OCD on SSRI	NA	risperidone (AT) aripiprazole (AT)	R: 1.7 mg (0.8) A: 8.9 mg (3.1)	R: 35/35 A: 34/34	R: 13.3 (2.2) A: 13.9 (2.5)	R: 94.3 A: 85.3	Change in illness severity and global functioning
Masi *et* *al.*	2010	USA	Case series	Treatment- resistant OCD on SSRI	Inpatients – 33.3%	aripiprazole (AT)	12.2 mg (3.4)	39/39	14.6 (1.2)^a^	71.8	Change in illness severity and global functioning
Thomsen et al.	2004	Denmark	Case series	Treatment- resistant OCD on SSRI	NA	risperidone (AT)	1.7 mg (0.4)	12/12	16.1 (0.9)	41.7	Change in OCD symptoms
Özdemir	2025	Turkey	Case series	Treatment- resistant OCD	NA	Amisulpride (AT)	100 mg	1/1	15	100	Change in OCD symptoms
**Mood dysregulation and borderline personality disorder**											
Mole *et* *al.*	2022	USA	Case series	Emerging BPD (1 patient on SSRI)	NA	lurasidone (AT & MT)	40 mg (0)	2/2	16.5 (0.5)	0	Improvement in disease- related symptoms
Pan et al.	2018	Taiwan	Open-label uncontrolled study	DMDD + ADHD & Methyl-phenidate medication	NA	aripiprazole (AT)	4.17 mg (1.20)	31/24	10.7 (2.37)	83.9	Change in clinical symptoms related to DMDD and ADHD
White et al.	2017	UK	Case series	Severe emerging BPD	Inpatients – 100%	clozapine (NA)	Mean + SD: N/A Range: 125-250 mg	15/15	16.5 (0.5)	0	Change in global functioning and rate of incidents
Krieger et al.	2011	Brazil	Open-label uncontrolled study	DMDD	Race: European-Brazilian: 66.7% Socio-econo-mic level of family: Middle upper: 47.6% Middle: 52.4%	risperidone (MT)	1.28 mg (0.58)	21/19	10.4 (2.8)	42.9	Change in irritability
Podobnik et al.	2011	Croatia	Open-label uncontrolled study	Develo-ping BPD	NA	quetiapine (AT)	389.8 mg (153.3)	22/22	15.4 (1.7)	50	Change in BPD related symptoms
**Depression**											
Moon et al.	2023	USA	Case series	MDD with psycho-tic features; IED and UMD; DMDD with psychotic features	Black – 25% Hispanic – 75%	aripiprazole (i.m. aripiprazole-lauroxil long-acting injection) (AT & MT)	MDD: 441 mg LAI IED: 882 mg LAI DMDD: 662 mg LAI	MDD: 2/2 IED: 1/1 DMDD: 1/1	MDD: 15.5 (1.5) IED: 15 DMDD: 15	MDD: 0 IED: 100 DMDD: 100	Readmission trend following initiation of injectable drug therapy
Yang et al.	2022	South Korea	Case report	A depressive mood, NSSI and suicidal ideation	NA	clozapine (MT)	25 mg	1/1	15	0	Improvement of NSSI and depressive symptoms
Pathak et al.	2005	USA	Case series	Treat-ment-resistant MDD	White – 100%	quetiapine (AT)	218.8 mg (49.6)	8/8	15 (1.1)^b^	37.5	Improvement in patients’ clinical picture

^a^Age range 12–18 years old; data from 18 years old patient(s) could not be separated.^b^Does not include one patient, whose age was not reported.NA = not available; OCD = obsessive-compulsive disorder; MDD = major depressive disorder; IED = intermittent explosive disorder; UMD = unspecified mood disorder; DMDD = disruptive mood dysregulation disorder; ADHD = attention-deficit hyperactivity disorder; BPD = borderline personality disorder; NSSI = non-suicidal self-injury; AT = augmentation therapy; MT = monotherapy; LAI = long-acting injectable

The studies were published between 2004 and 2025 (mean publication year was 2015). Two studies were from the United States, two from Italy, two from Turkey, and the remaining seven studies originated from Australia, Brazil, Croatia, Denmark, England, South Korea, and Taiwan. Treatment durations ranged from four weeks to 18 months across studies. The age of patients ranged from six to 18 years, since it is likely that one 18-year-old could not be excluded from one of the studies [21]. The mean age of the patients was 13.5 years.

Differences in clinical response between child and adolescent populations could not be assessed due to the lack of individual data. Neither a meta-analysis nor a publication bias assessment was performed since our predefined criteria (see Methods section) were not met.

### Quality assessment

**Fig. 2:**
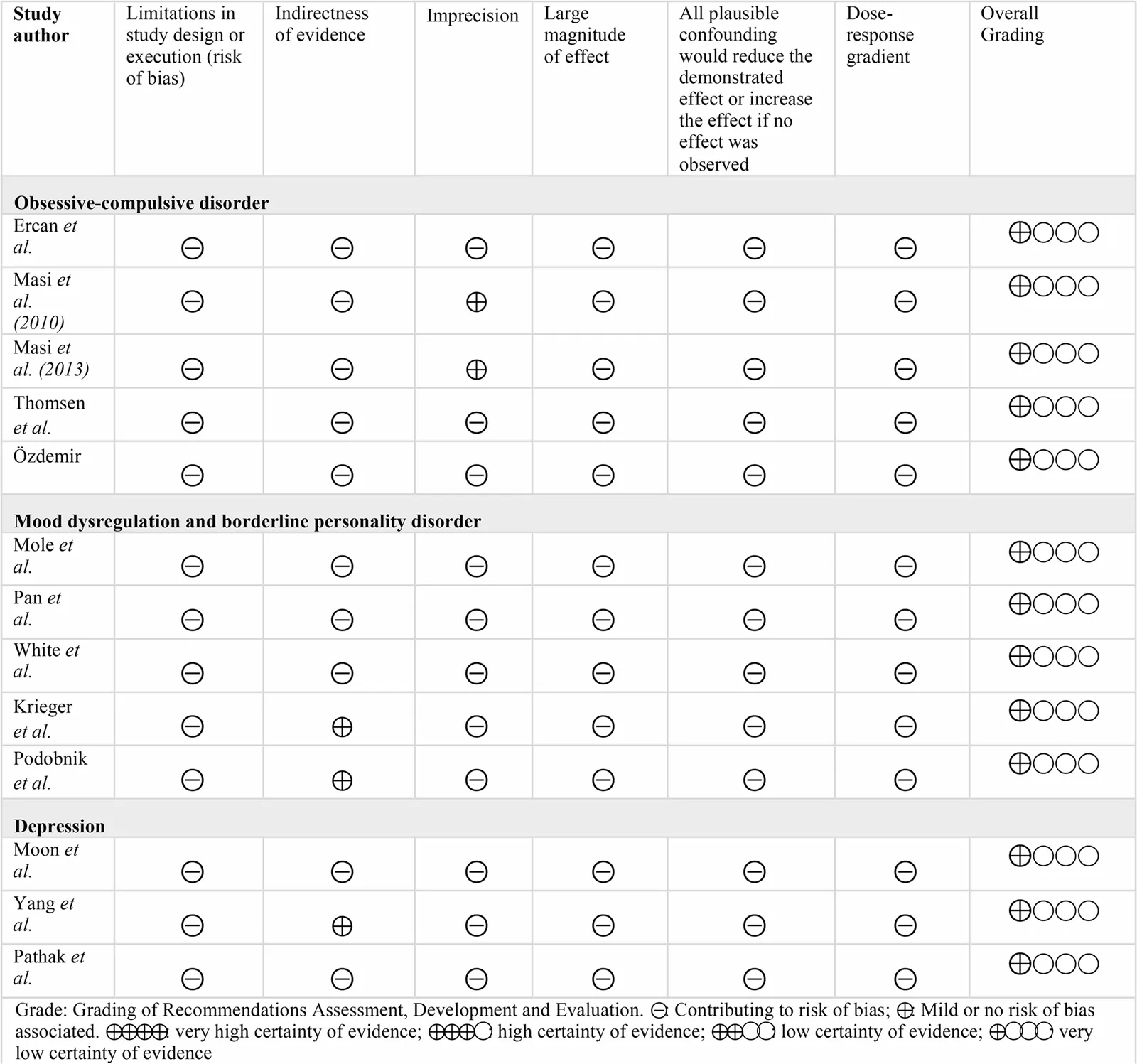
Methodological quality assessment for included studies using GRADE criteria

An overview of the quality assessment in accordance with the GRADE criteria is provided in Fig. 2. All 13 of the included studies were observational studies with no placebo control. None of the studies demonstrated a large magnitude of effect, a dose-response gradient, nor did they reliably control for confounding factors. Furthermore, all studies had additional concerns, such as imprecision due to low sample size and indirectness of evidence (concomitant use of multiple other medications and patients with varying comorbidities), as well as a high risk of bias due to unreliable outcome measurements (some studies used only self-reported measures instead of standardized symptom scales). Inconsistency of results was not assessed due to a lack of comparable study settings. Similarly, publication bias could not be assessed through a funnel plot due to a lack of studies with similar outcome measures. The overall rating for all the included studies was deemed as ‘very low’ certainty of evidence. This means that there is a high level of uncertainty in the estimations of effect from these studies.

### Treatment effects of antipsychotic use by disorder

**Table 2:** Study results

Study author	Study population	Intervention (I), control (C); number of patients (*n*)	Study duration	Outcome measures of interest^a^	Baseline, mean ± SD	Endpoint, mean ± SD	Change in raw mean scores	Change in standardized means between baseline and endpoint^b, c^
**Obsessive-compulsive disorder**								
Ercan et al.	Treatment-resistant OCD	I: aripiprazole (16) C: NA	12 weeks	Obsessive-compulsive symptoms (CY-BOCS)	30.6 ± 5.7	15.3 ± 7.0	-15.3	2.4
Masi et al.	Treatment-resistant OCD	I: risperidone (35) C: aripiprazole (34)	12 weeks	Severity of symptoms (CGI-S)	I: 5.6 ± 0.7 C: 5.6 ± 0.8	I: 3.4 ± 0.9 C: 3.0 ± 0.9	I: -2.2 C: -2.6	I: 2.7 C: 3.1
				Improvement in symptoms (CGI-I)	I: NA C: NA	I: 2.4 ± 0.7 C: 2.3 ± 0.6	I: NA C: NA	I: NA C: NA
				Overall functioning (CGAS)	I: 40.3 ± 5.8 C: 41.0 ± 4.9	I: 53.3 ± 10.5 C: 54.9 ± 9.6	I: +13.0 C: +13.9	I: 1.5 C: 1.8
Masi *et* *al.*	Treatment-resistant OCD	I: aripiprazole (39) C: NA	6 months	Severity of symptoms (CGI-S)	6.0 ± 0.9	3.5 ± 1.0	-2.5	2.6
				Improvement in symptoms (CGI-I)	NA	2.3 ± 0.5		
				Overall functioning (CGAS)	39.2 ± 5.8	49.0 ± 9.2	+ 9.8	1.3
Thomsen et al.	Treatment-resistant OCD	I: risperidone (12) C: NA	12 weeks	Overall functioning (CGAS)	68.3 ± 12.0	73.3 ± 9.9	+ 5.0	0.5
				Obsessive-compulsive symptoms (CY-BOCS)	23.4 ± 2.0	19.4 ± 3.0	-4.0	1.5
Özdemir	Treatment-resistant OCD	I: amisulpride (1) C: NA	3 months		NA	NA	NA	NA
**Mood dysregulation and borderline personality disorder**								
Mole *et* *al.*	Emerging BPD	I: lurasidone (2) C: NA	Patient specific (4 weeks to 18 months)		NA	NA	NA	NA
Pan et al.	DMDD + ADHD	I: aripiprazole (24) C: NA	6 weeks	BYI-II (angry)^d^	59.6 ± 9.2	53.2 ± 7.9	-6.4	0.7
				BYI-II (disruptive behavior)^d^	60.2 ± 8.9	54.0 ± 6.9	-6.2	0.8
				CBCL (anxious/depressed)^d^	64.8 ± 9.4	58.1 ± 7.8	-6.7	0.8
				CBCL (social problems)^d^	68.9 ± 10.8	61.9 ± 8.6	-7.0	0.7
				CBCL (rule-breaking behavior)^d^	66.9 ± 6.6	58.9 ± 8.0	-8.0	1.1
				CBCL (aggressive behavior)^d^	71.3 ± 7.7	60.3 ± 8.1	-11.0	1.4
White et al.	Severe emerging BPD	I: clozapine (15) C: NA	24 weeks	Overall functioning (CGAS)	12.0, SD not reported	24.4, SD not reported	+ 12.4	NA
Krieger et al.	DMDD	I: risperidone (19) C: NA	8 weeks	Irritability (ABC-I)	25.9 ± 12.6	11.3 ± 13.3	-14.6	1.1
				Severity of symptoms (CGI-S)	4.5 ± 0.5	2.6 ± 0.6	-1.9	3.4
				Overall functioning (CGAS)	46.9 ± 5.9	70.1 ± 8.2	+ 23.2	3.2
				Depressive symptoms (CDRS)	34.3 ± 7.0	22.5 ± 3.4	-11.8	2.1
				Anxious symptoms (SCARED)	34.7 ± 19.4	21.0 ± 18.8	-13.7	0.7
Podobnik et al.	Developing BPD	I: quetiapine (22) C: NA	24 weeks	Depressive symptoms (KADS-6)	11.9 ± 2.9	7.9 ± 3.4	-4.0	1.3
				Overall aggression (OAS-M)	50.6 ± 25.1	35.7 ± 26.4	-14.9	0.6
				OAS-M (aggression)^d^	39.0 ± 22.5	29.0 ± 23.1	-10.0	0.4
				OAS-M (irritability)^d^	7.8 ± 1.3	5.9 ± 2.3	-1.9	1.0
				OAS-M (suicidality)^d^	3.9 ± 4.2	0.9 ± 2.6	-3.0	0.9
				Overall functioning (CGAS)	32.5 ± 11.9	44.5 ± 14.9	+ 12.0	0.9
**Depression**								
Moon et al.	MDD with psychotic features; IED and UMD; DMDD with psychotic features	I: aripiprazole (i.m. long-acting injection) (4) C: NA	Not disclosed		NA	NA	NA	NA
Yang et al.	A depressive mood, NSSI and suicidal ideation	I: clozapine (1) C: NA	4 weeks	Depressive symptoms (HDRS)	28	13	-15	NA
Pathak et al.	Treatment-resistant MDD	I: quetiapine (8) C: NA	Patient specific (4–16 weeks)	Severity of symptoms (CGI-S)	5.4 ± 0.9	NA	NA	NA
				Improvement in symptoms (CGI-I)	NA	1.8 ± 0.8	NA	NA

^a^See Supplement 4, available online, for score ranges for all symptom scales and guidelines for score interpretation.

^b^See Supplement 5, available online, for the equation showing, how Cohen’s *d*_av_ was calculated.

^c^Positive values indicate change in desirable direction.

^d^Individually analyzed subscale.

OCD = obsessive-compulsive disorder; MDD = major depressive disorder; IED = intermittent explosive disorder; UMD = unspecified mood disorder; DMDD = disruptive mood dysregulation disorder; ADHD = attention-deficit hyperactivity disorder; BPD = borderline personality disorder; NSSI = non-suicidal self-injury.

NA = not applicable; (C)Y-BOCS = Children’s Yale-Brown Obsessive Compulsive Scale; CGI-S = Clinical Global Impression - Severity; CGI-I = Clinical Global Impression - Improvement; CGAS = Children’s Global Assessment Scale; BYI-II = Beck Youth Inventory 2nd edition; CBCL = Child Behavior Checklist; ABC-I = Aberrant Behavior Checklist Irritability subscale; CDRS = Children’s Depression Rating Scale; SCARED = Screen for Child Anxiety Related Disorders; KADS-6 = 6-item Kutcher Adolescent Depression Scale; OAS-M = The Overt Aggression Scale Modified; HDRS = Hamilton Depression Rating Scale.

### Obsessive-compulsive disorder

We identified five studies on APs for treatment-resistant OCD. One of the studies was an open-label clinical trial [22], one was a cohort study with two treatment groups [23], and the remaining three were case series.[21; 24; 25] None of the studies were placebo-controlled. Four of the five studies examined APs as an augmentation for SSRI therapy. The studies included 137 patients, of whom 47 received risperidone, and 89 received aripiprazole. In addition, one patient received amisulpride.

SSRI augmentation with aripiprazole was examined in one case series (*n* = 39) [21] and one cohort study with two treatment groups (*n* = 34) [23], both by Masi et al. The patients in these two studies were derived from the same population; therefore, some patients might have been included in both. However, this was not reported. These studies included patients with comorbid disorders (some patients with bipolar disorder could not be excluded), use of other concomitant treatment options (cognitive-behavioral therapy and simultaneous medications), and lack of OCD-specific measurements. Both studies used illness severity and change in overall social functioning as outcomes. Positive changes in the mean total scores were noted on both clinician-rated standardized symptom scales. Across the two studies, 44 of the total 73 participants were deemed responders regarding their OCD symptoms. Both studies also stated that responders’ social functioning was superior at baseline when compared to non-responders. Interestingly, a corresponding trend in illness severity was noted only in one of the studies [23]. 

SSRI augmentation with risperidone was examined in two studies – a cohort study with two treatment groups by Masi et al. (*n* = 35) [23] and a case series by Thomsen (*n* = 12) [24]. Both studies utilized a clinician-rated symptom scale for measuring functioning in various social areas. All patients in the study by Masi et al. had a comorbid tic disorder, while a single patient in the study by Thomsen had a comorbid autism spectrum disorder. Masi et al. had clinicians rate illness severity, while Thomsen had clinicians rate the severity of obsessive and compulsive symptoms, as well. When compared to baseline, symptom scores were lower and functioning scores were higher (see Table 2.), but their clinical significance remains unclear due to the nature of these studies. Masi et al. reported no statistically significant difference between the choice of drug therapy (aripiprazole or risperidone augmentation).

An open-label, uncontrolled trial by Ercan et al. (*n* = 16) focused on aripiprazole monotherapy in the treatment of treatment-resistant OCD [22]. Patients in this study had various comorbidities with attention-deficit/hyperactivity disorder (ADHD) being the most prevalent (eight out of 16 patients). None of the patients had comorbid psychotic disorders. Around 50% reduction in mean score on a clinician-rated OCD symptom scale was reported (see Table 2.), going from severe to mild-moderate symptom severity. On another clinician-rated scale, symptom severity was reported to have improved significantly or more in 13 of the 16 patients. Interestingly, this study used much smaller doses of aripiprazole than those used in other studies for augmentation purposes.

Lastly, one case series by Özdemir with one patient meeting our inclusion criteria was identified [25]. In this study, the patient received amisulpride as augmentation therapy for SSRI in the treatment of treatment-resistant OCD. The patient had previously been prescribed aripiprazole and risperidone, both of which caused excessive adverse effects and insufficient treatment response. The patient’s OCD symptoms were reported to have significantly improved during his 3-month follow-up period, and he continues using amisulpride. No clinical symptom scales were used in this study.

### Mood dysregulation and borderline personality disorder

We identified six studies that examined APs in the treatment of disruptive mood dysregulation disorder, emerging borderline personality disorder, and other disorders related to unstable mood regulation in adolescents. Three of these were open-label trials, and three were case series. None of the studies utilized a placebo control group. The studies included a total of 98 patients. APs used in these studies were aripiprazole, risperidone, quetiapine, clozapine, and lurasidone. Of these, aripiprazole, risperidone, and lurasidone were used as monotherapy, while aripiprazole, quetiapine, clozapine, and lurasidone were used as augmentation for other drug therapies, as specified later. As seen in Table 2., the outcomes varied across studies; however, clinician-rated overall social functioning was used in three of the studies.

An open-label trial by Krieger et al. studying risperidone as monotherapy included 21 patients with disruptive mood dysregulation disorder, of whom 19 completed the treatment [26]. Comorbid disorders were prevalent and included namely ADHD (*n* = 15), oppositional defiant disorder (*n* = 17), and anxiety disorders (*n* = 15). Positive changes in standardized means were observed on all symptom scales used, with parent-rated severity of irritability being the primary outcome. By the end of the eight-week study, the mean severity of irritability had decreased from moderate to low, while mean overall social functioning had improved from moderate/severe to slight impairment.

Aripiprazole was examined in two studies. Pan et al. assessed it as augmentation therapy with methylphenidate for patients with disruptive mood dysregulation disorder and a comorbid ADHD in an open-label trial including 31 patients, of whom 24 completed their treatment [27]. Treatment response was measured by assessing change in (1) parent-reported ADHD symptoms, (2) parent-reported internalizing and externalizing symptoms, and (3) self-reported symptoms of depression, anxiety, self-concept, disruptive behavior, and anger, all of which were assessed both before and after augmentation. Aripiprazole augmentation was associated with reductions in mean scores on all but one of the subscales that were associated with disruptive mood dysregulation disorder. See Table 2. for a breakdown of results for individual subscales.

The other study on aripiprazole was a case series by Moon et al. with two patients who used a prodrug of aripiprazole as monotherapy for a variety of mood dysregulation-related indications [28]. Families of both patients reported improvements in the patients’ symptoms, and both continued their treatment after being initially discharged from the treatment facility. This study presented equivocal evidence for the efficacy of aripiprazole in the treatment of unstable mood, as it did not utilize any clinical symptom scales to assess the treatment response.

One pre-post trial by Podobnik et al. examined augmentation of SSRI therapy with quetiapine [20]. The trial included 22 adolescent patients with symptoms corresponding to adult borderline personality disorder. This study excluded patients with depressive, psychotic, or eating disorders, as well as those with other concomitant medications than SSRIs. No individual data is available, e.g., specific comorbidities. The patients were examined before, during, and after quetiapine intervention. The measures included clinician-rated overall social functioning, patient-rated depressive symptoms, and clinician-rated severity of current aggressive acts. Improvements in both raw and standardized mean scores were observed on all clinical scales used after the 24-week treatment period.

One retrospective study by White et al. assessed clozapine in the treatment of severe emerging borderline personality disorder [29]. This study included a total of 15 inpatients from two different treatment facilities. Some patients used other concurrent medications, including mood stabilizers and antidepressants. Improvements in clinician-rated overall social functioning were noted on 14 of the 15 patients, with mean scores changing from 12.7 to 24.4; however, no standard deviations were reported. In addition to overall social functioning, the number of incidents associated with inpatient care, such as restraining, seclusion, and deliberate self-harm, was reported to have decreased after initiation of clozapine treatment.

Lastly, lurasidone was used in one study by Mole et al. including two patients with emerging borderline personality disorder [30]. In retrospective case note inspections, some positive effects were observed in one of the patients during an 18-month treatment period, while the clinical response for the other patient remained unclear during her 4-week treatment period. No use of clinical symptom scales was reported.

### Depression

As described in Table 1, we identified three studies on the efficacy of APs in the treatment of depressive symptoms. Two of them were case series, which had studied quetiapine and aripiprazole as adjunctive therapy for SSRIs in the treatment of major depressive disorder. The third was a case report of a patient using clozapine monotherapy for depressive mood and suicidal ideation. These studies included a total of 11 patients, of whom eight were treated with quetiapine.

In a case series by Pathak et al., quetiapine was studied as an adjunctive treatment for SSRIs in patients with depression [31]. Eight patients met our inclusion criteria. All of the patients had comorbid diagnose(s), with ADHD being the most prevalent (*n* = 6). Treatment duration varied from four to 16 weeks between the patients. Six patients were deemed responders on a clinician-rated symptom scale that measured improvement in patients’ clinical picture.

The case series by Moon et al. included two patients with major depressive disorder that received aripiprazole long-acting injectable [28]. Evidence for the efficacy of aripiprazole as adjunctive treatment remained equivocal, as the study only included information on the continuance of treatment and/or changes made to it. The study reported no changes in symptoms by patients, family, or professionals in these cases. Of the two patients of interest, one was reinitiated on the same therapy after 10 months, while the other had her medication changed and aripiprazole treatment ceased due to poor response.

In a case report by Yang et al., low-dose clozapine monotherapy lowered the single patient’s score on a clinician-rated symptom scale from severe to mild depression during her four-week-long treatment period [32]. 

### Anxiety

No studies of antipsychotic treatment for anxiety in children or adolescents were identified. However, the study by Pan et al., including patients with both disruptive mood dysregulation disorder and ADHD, reported positive changes on both parent-rated and patient-reported anxiety-related subscales [27]. It remains unclear whether these changes were a result of the possible anxiolytic effects of aripiprazole or if the patients, for example, felt less anxious following relief of the symptoms that were the main target of the treatment.

### Sleep disorders

No studies on antipsychotic treatment for sleep disorders in children or adolescents were identified.

## Discussion

In this systematic review, we have provided a comprehensive overview of the full range of studies researching APs among children and adolescents in common off-label indications. All 13 studies included in this review were individually assessed according to the GRADE quality assessment protocol and provided “very low” level of evidence. Study limitations included small sample sizes, lack of control groups, and several other weaknesses contributing to the risk of bias. Therefore, there is high uncertainty regarding the correctness of the estimates of effect from these studies, and no conclusions for the efficacy of AP use in adolescents can be established. The very low level of evidence found for off-label AP use in children and adolescents presents a sharp distinction to current prescription practices.

Despite the low level of evidence, we identified several results that support doing further research. In the limited number of studies on SSRI augmentation for treatment-resistant OCD, patients’ scores on clinical symptom scales improved after augmenting SSRIs with aripiprazole or risperidone.[21; 23; 24] For example, changes in standardized means between baseline and endpoint were in the range of 0.5 to 1.8 when measured on the Children’s Global Assessment Scale. Similar results were reported for aripiprazole monotherapy [22]. However, as noted above these studies were uncontrolled and had a plethora of other limitations. Without a reliable control arm the results may be due to the fluctuation in the natural course of psychiatric disorders or even selective reporting [33], though response to the drug therapy can not be excluded. This should be researched further in future methodologically sound placebo-controlled studies.

The evidence for unstable mood-related disorders and major depressive disorder was also very low. Symptom reductions on clinical rating scales were observed following treatment with risperidone [26], quetiapine [20], and aripiprazole [27], in populations with unstable mood-related disorders. The changes in core symptoms between baseline and endpoint ranged from 0.6 to 1.4 in standardized mean scores (see Table 2.),[20; 27] which would be clinically meaningful if there had been a control intervention alongside methodologically stronger study designs.

Our findings align with previous studies. A systematic review from 2017 noted the lack of high-quality studies on AP use in the treatment of depression, OCD, and behavioral problems in children and adolescents [7]. A research review from 2020 reported a lack of randomized controlled trials on the efficacy of APs as augmentation for the treatment of treatment-resistant major depressive disorder [34], while a 2017 systematic review identified only two studies not meeting our exclusion criteria that researched AP efficacy in the treatment of severe mood dysregulation and disruptive mood dysregulation disorder in children and adolescents [35]. One of these studies was included in our research [26], while the other was terminated due to a lack of participants (NCT02063945). Likewise, an expert opinion from 2018 noted that no studies had evaluated the efficacy of APs in adolescent populations in the treatment of generalized anxiety disorder [36], though many studies in adult populations using different 2nd generation APs exist.[37; 38].

The low level of evidence for AP use in adolescents explains why clinical practice guidelines do not mention APs at all or recommend a careful approach and short treatment duration when prescribed.[11; 12; 15] Although we examined a broader range of off-label indications and study types, which provides a more comprehensive understanding of the current state of research, our findings are consistent with previous studies and offer no reason to alter these guidelines.

As the current evidence for off-label AP use in children and adolescents does not support today’s prescription practices, it is worthwhile to examine the evidence in adult populations. A network meta-analysis from 2019 noted that in adults, only aripiprazole provided meaningfully better treatment results than placebo in patients with OCD, when studies with high risk of bias were excluded [39]. An umbrella review from 2024 observed that quetiapine seemed effective in reducing risk of recurring episodes in the treatment of generalized anxiety disorder, but long-term efficacy remained unclear [17]. A 2019 systematic review on adult depression inspected augmentation of SSRI treatment with various 2nd generation APs, including cariprazine, ziprasidone, quetiapine, and olanzapine [40]. In this review, depressive symptoms were reported to have decreased after augmentation in the included studies. The total number of studies examining APs was low (k = 7), and therefore, the level of evidence remained primarily low or moderate for AP interventions. For borderline personality disorder, a 2022 systematic review reported AP treatment to have little to no effects on symptom severity, though the certainty of evidence remained very low [41]. A systematic review in 2022 found that quetiapine significantly improved sleep quality in both healthy subjects and patients with generalized anxiety disorder and/or major depressive disorder [42]. However, the long-term efficacy and safety remain unclear. Whilst the evidence for AP use in adult populations is low to moderate for some indications, it seems to provide benefits when compared to placebo. These findings might have influenced clinical practice and encouraged AP use among adolescents.

An examination of prescribing trends and the strength of evidence regarding off-label antipsychotic use in both adult and pediatric populations suggests that evidence derived from adult studies has likely been extrapolated to inform pediatric practices or clinicians have drawn their own conclusions about efficacy [43]. Given the scarcity of randomized controlled trials in child and adolescent populations and the overall limited evidence base, overreliance on adult data is likely present, which is also reflected in some guidelines [15]. It is important to note that adult studies are not directly applicable to children and adolescents due to differences in psychiatric symptom presentation, neurobiology, pharmacodynamics, and pharmacokinetics that impact metabolic side effects.[8; 43].

On the other hand, relying on lower-quality pediatric studies is not suitable either. All except one study in our systematic review were uncontrolled studies, and none aimed to robustly address confounding by indication. These studies can not account for the significant variability of disorder trajectories in adolescent psychiatric disorders and can therefore not conclude whether the results are due to medication or spontaneous recovery. While RCTs are the gold standard for causal inference and for example a promising protocol for an RCT on adolescents with sleeping disorders has been published [44], also other study types to consider do exist. Controlled observational cohort studies with target trial emulation protocols and robust causal inference methods, such as instrumental variable approaches, have the possibility to improve causal inference. Such studies have been published upon other topics within psychiatry [45–47], and with the rise of large-scale electronic health records and improved statistical methods, they provide a promising approach along RCTs. Another advantage of using real-worĺd data is the inclusion of all ethnic and sociodemographic populations and/or minorities that enter health services, but often go underrepresented in trials.

Our study has several limitations, of which many are intrinsic to literature searches. It is possible that some relevant studies were excluded because they were not published in English. Most of the data was derived from published uncontrolled trials or case series. These study designs are not sufficient for drawing any valid conclusions on treatment efficacy. Studies included in our review could have overestimated the changes in symptom scores between baseline and endpoint due to natural fluctuations in symptom presentations and the fact that small trials rarely report negative findings. The quality of evidence was defined as “very low” for all included studies, severely limiting our capability to draw conclusions on efficacy. Our included studies were also very heterogeneous. This heterogeneity was especially related to comorbidities in study populations, varying outcome measures used in different studies, and study designs. Publication bias could not be assessed due to the low number of studies with comparable outcomes, and meta-analyses were not performed due to a lack of comparative data.

## Conclusions

Our systematic review found very low level of evidence for the off-label use of antipsychotics in disorders relating to depression, anxiety, sleep, mood dysregulation, and OCD in children and adolescents. While high-quality comparative studies on AP use are urgently needed, this review provides the most up-to-date and comprehensive review of AP use among children and adolescents for these common off-label indications. This is especially important due to the high prevalence of these disorders. We acknowledge the need to offer severely ill young patients effective treatment when on-label treatment options do not relieve symptoms enough for the individual patient. However, this study highlights the need to carefully weigh the benefits and harms when prescribing APs to children and adolescents. The lack of efficacy data and known metabolic, cardiovascular, and neurological risks of APs do not support the current rising trends in AP use among adolescents.

### Data availability

Not applicable.

### Code availability (software application or custom code)

Not applicable.

## Supplementary Information

Below is the link to the electronic supplementary material.

Supplementary Material 1

Supplementary Material 2

Supplementary Material 3

Supplementary Material 4

Supplementary Material 5

## Data Availability

Not applicable.
