# Supplementary material for: Systematic review: Efficacy of off-label antipsychotic use in children and adolescents with obsessive-compulsive, mood dysregulation, depression, anxiety, and sleep-related disorders
Source: Eur Child Adolesc Psychiatry. 2026 May 18;35(8):2367–81. doi: 10.1007/s00787-026-03049-9 (PMC13652945; doi:10.1007/s00787-026-03049-9)
Supplement: Supplementary file 1 [file 787_2026_3049_MOESM1_ESM.odt]

Systematic review: Efficacy of off-label antipsychotic use in children and adolescents with obsessive-compulsive, mood dysregulation, depression, anxiety, and sleep-related disorders
European Child + Adolescent Psychiatry

Authors: Akseli K. Holopainen, MS*,a; Pyry A. Vainiotalo, MS*,a; Bianca P. Arrhenius, MD, PhDb,c; Ida Ringbom, MD, PhDd; Mirja Laurila, MD, Phdd; David Gyllenberg, MD, PhDd
(*) Shared first authorship  
a University of Helsinki, Faculty of Medicine  
b Finnish Medical Society Duodecim, Helsinki, Finland 
c Department of General Practice and Primary Health Care, University of Helsinki  
d Department of Adolescent Psychiatry, Helsinki University Hospital, Helsinki, Finland

Corresponding author: David Gyllenberg. 
Email: david.gyllenberg@hus.fi
ORCID: 0000-0003-3220-9805


Online resource 4 Score ranges for clinical symptom scales used in the included studies and guidelines for score interpretation
Name of scale
Measurement
Range
Bottom grade explanation
Middle grade explanation
Top grade explanation
(C)Y-BOCS1
Obsessive and compulsive symptoms
0-40
0-7 = subclinical
16-23 = moderate symptoms
32-40 = extreme symptoms
CGI-S2
Severity of psychiatric illness
1-7
1 = normal, not at all ill
4 = moderately ill
7 = among the most extremely
ill patients
CGI-I2
Improvement in illness presentation
1-7
1 = very much improved since the
initiation of treatment
4 = no change from baseline (the
initiation of treatment)
7 = very much worse since the
initiation of treatment
CGAS3
General functioning of adolescent patient
1-100
1-10 =Needs constant supervision (24 hour care)
41-50 = Moderate degree of interference in functioning in most social areas or severe impairment of functioning in one social area;
51-60 = Variable functioning with sporadic difficulties or symptoms in several but not all social areas
91-100 = Superior functioning
BYI-II (T-
score)4
Symptoms of depression, anxiety, anger, disruptive behavior, and self-concept
1-100
≤ 55 = average
60-69 = moderately elevated
70 ≤ = extremely elevated
CBCL (t-
score)5
Behavioral and emotional problems
1-100
< 65 ≈ normal range
65-70 ≈ borderline range
70 < ≈ clinical range
ABC-I*
Symptoms of heightened irritability
0-45
0-10 = Minimal or no irritability
20-30 = Moderate irritability
30-45 = Severe irritability
CDRS6
Depressive symptoms
17-113
≤ 28 = minimal or no depressive
symptoms
29-39 = Some depressive symptoms
40 ≤ = Indicative of
depression
SCARED7
Symptoms of anxiety
0-82
< 25 = Indicative of no present
anxiety disorder

25 ≤ = Indicative of a present
anxiety disorder
KADS-68
Depressive symptoms
0-18
0-5 = probably not depressed

6 ≤ = Possible depression
OAS-M9
Recent aggressive behavior
0-40
0-10 = None or mild aggression in subcategories (Verbal aggression, aggression against property, autoaggression, physical aggression)

30-40 = Severe or extreme aggression in multiple subcategories (Verbal aggression, aggression against property, autoaggression,
physical aggression)
HDRS10
Depressive symptoms
0-52
0-7 = normal 
17-23 = moderate depression 
24 < = severe depression 

*Consists of 15 items, each rated from 0 (no problems) to 3 (severe problem), which are then added up to form a total score of 0-45.11
Supplemental reference list
1.	Goodman WK, Price LH, Rasmussen SA. CY-BOCS Severity Ratings [PDF]. Published online September 1997. Accessed October 26, 2025. https://gtw-health.com/wp-content/uploads/2018/03/Yale-Brown-Severity-Ratings.pdf
2.	Busner J, Targum SD. The Clinical Global Impressions Scale. Psychiatry Edgmont. 2007;4(7):28-37.
3.	Shaffer D, Gould MS, Brasic J, et al. A Children’s Global Assessment Scale (CGAS). Arch Gen Psychiatry. 1983;40(11):1228-1231. doi:10.1001/archpsyc.1983.01790100074010
4.	Beck JS, Beck AT, Bolly JB. BYI-2 Score Report Sample [PDF]. Published online 2016. Accessed October 26, 2025. https://www.pearsonclinical.co.uk/content/dam/school/global/clinical/us/assets/beck/BYI-2-Score-Report-Sample.pdf
5.	Pandolfi V, Magyar CI, Dill CA. An Initial Psychometric Evaluation of the CBCL 6–18 in a Sample of Youth with Autism Spectrum Disorders. Res Autism Spectr Disord. 2012;6(1):96-108. doi:10.1016/j.rasd.2011.03.009
6.	Mayes TL, Bernstein IH, Haley CL, Kennard BD, Emslie GJ. Psychometric Properties of the Children’s Depression Rating Scale–Revised in Adolescents. J Child Adolesc Psychopharmacol. 2010;20(6):513-516. doi:10.1089/cap.2010.0063
7.	Birmaher B, Brent DA, Chiappetta L, Bridge J, Monga S, Baugher M. Psychometric Properties of the Screen for Child Anxiety Related Emotional Disorders (SCARED): A Replication Study. J Am Acad Child Adolesc Psychiatry. 1999;38(10):1230-1236. doi:10.1097/00004583-199910000-00011
8.	Appendix 3: 6-Item Kutcher Adolescent Depression Scale (KADS). In: Suicide Risk Management. John Wiley & Sons, Ltd; 2007:119-123. doi:10.1002/9780470750933.app3
9.	American Academy of Pediatrics. Modified Overt Aggression Scale (MOAS) [PDF]. Published online 2010. Accessed October 26, 2025. https://depts.washington.edu/dbpeds/Screening%20Tools/Modified-Overt-Aggression-Scale-MOAS.pdf 
10.	Zimmerman M, Martinez JH, Young D, Chelminski I, Dalrymple K. Severity classification on the Hamilton depression rating scale. J Affect Disord. 2013;150(2):384-388. doi:10.1016/j.jad.2013.04.028
11.	Kildahl AN, Hellerud JMA, Halvorsen MB, Helverschou SB. The Aberrant Behavior Checklist in a Clinical Sample of Autistic Individuals with Intellectual Disabilities and Co-Occurring Mental Health Problems: Psychometric Properties, Factor Structure, and Longitudinal Measurement Invariance. J Autism Dev Disord. Published online January 4, 2025. doi:10.1007/s10803-024-06697-5
