# Supplementary material for: Systematic review: Efficacy of off-label antipsychotic use in children and adolescents with obsessive-compulsive, mood dysregulation, depression, anxiety, and sleep-related disorders
Source: Eur Child Adolesc Psychiatry. 2026 May 18;35(8):2367–81. doi: 10.1007/s00787-026-03049-9 (PMC13652945; doi:10.1007/s00787-026-03049-9)
Supplement: Supplementary file 2 [file 787_2026_3049_MOESM2_ESM.odt]

Systematic review: Efficacy of off-label antipsychotic use in children and adolescents with obsessive-compulsive, mood dysregulation, depression, anxiety, and sleep-related disorders
European Child + Adolescent Psychiatry

Authors: Akseli K. Holopainen, MS*,a; Pyry A. Vainiotalo, MS*,a; Bianca P. Arrhenius, MD, PhDb,c; Ida Ringbom, MD, PhDd; Mirja Laurila, MD, Phdd; David Gyllenberg, MD, PhDd
(*) Shared first authorship  
a University of Helsinki, Faculty of Medicine  
b Finnish Medical Society Duodecim, Helsinki, Finland 
c Department of General Practice and Primary Health Care, University of Helsinki  
d Department of Adolescent Psychiatry, Helsinki University Hospital, Helsinki, Finland

Corresponding author: David Gyllenberg. 
Email: david.gyllenberg@hus.fi
ORCID: 0000-0003-3220-9805


Online resource 5 Formula, which was used to calculate standardized mean difference (applied from formula for calculating Cohen’s dav)

, where SMD is standardized mean difference, M is the mean score and SD is the standard deviation of scores for the study population.
