# Supplementary material for: Systematic review: Efficacy of off-label antipsychotic use in children and adolescents with obsessive-compulsive, mood dysregulation, depression, anxiety, and sleep-related disorders
Source: Eur Child Adolesc Psychiatry. 2026 May 18;35(8):2367–81. doi: 10.1007/s00787-026-03049-9 (PMC13652945; doi:10.1007/s00787-026-03049-9)
Supplement: Supplementary file 3 [file 787_2026_3049_MOESM3_ESM.odt]

Systematic review: Efficacy of off-label antipsychotic use in children and adolescents with obsessive-compulsive, mood dysregulation, depression, anxiety, and sleep-related disorders
European Child + Adolescent Psychiatry

Authors: Akseli K. Holopainen, MS*,a; Pyry A. Vainiotalo, MS*,a; Bianca P. Arrhenius, MD, PhDb,c; Ida Ringbom, MD, PhDd; Mirja Laurila, MD, Phdd; David Gyllenberg, MD, PhDd
(*) Shared first authorship  
a University of Helsinki, Faculty of Medicine  
b Finnish Medical Society Duodecim, Helsinki, Finland 
c Department of General Practice and Primary Health Care, University of Helsinki  
d Department of Adolescent Psychiatry, Helsinki University Hospital, Helsinki, Finland

Corresponding author: David Gyllenberg. 
Email: david.gyllenberg@hus.fi
ORCID: 0000-0003-3220-9805


Supplementary table 1a Table showing indications for 2nd generation APs approved by European Medicines Agency in both children and adults 
Drug name
Indications in adolescent populations
Indications in adult populations
Notes
Amisulpride
NA
NA
Differences in national indications; no recommendation has been issued by EMA
Aripiprazole
Schizophrenia (≥15 years old)
Schizophrenia


Bipolar 1 disorder (≥13 years old)
Bipolar 1 disorder

Asenapine
NA
Bipolar 1 disorder

Brexpiprazole
Schizophrenia (≥13 years old)
Schizophrenia

Cariprazine
NA
Schizophrenia

Clozapine
NA
Treatment-resistant schizophrenia

Iloperidone
NA
NA
No EU marketing authorization
Lurasidone
Schizophrenia (≥13 years old)
Schizophrenia

Lumateperone
NA
NA
No EU marketing authorization
Olanzapine
NA
Schizophrenia


NA
Bipolar 1 disorder

Paliperidone
Schizophrenia (≥15 years old)
Schizophrenia


 Schizoaffective disorder

Pimavanserin
NA
NA
No EU marketing authorization
Quetiapine
NA
Schizophrenia


Bipolar 1 disorder


Adjunctive treatment for major depressive disorder

Risperidone
Persistent aggression associated with conduct disorders
Schizophrenia


Bipolar 1 & 2 disorders


Persistent aggression associated with Alzheimer’s disease

Ziprasidone
NA
Schizophrenia


Bipolar 1 disorder


Supplementary table 1b Table showing FDA-approved indications for 2nd generation APs in both children and adults1
Drug name
Indications in adolescent populations
Indications in adult populations
Amisulpride
NA
Post-operative nausea and vomiting
Aripiprazole
Schizophrenia (≥13 years old)
Schizophrenia

Bipolar 1 disorder (≥10 years old)
Bipolar 1 disorder

Tourette’s disorder (≥6 years old)
Adjunctive therapy for major depressive disorder

Symptoms of autism spectrum disorders (≥6 years old)

Asenapine
Bipolar 1 disorder (≥10 years old)
Schizophrenia


Bipolar 1 disorder
Brexpiprazole
Schizophrenia (≥13 years old)
Schizophrenia


Adjunctive therapy for major depressive disorder


Agitation associated with Alzheimer’s disease
Cariprazine
NA
Schizophrenia


Bipolar 1 disorder


Adjunctive therapy for major depressive disorder
Clozapine
NA
Treatment resistant schizophrenia


Suicidal ideation and behaviour associated with
schizophrenia and similar disorders
Iloperidone
NA
Schizophrenia
Lurasidone
Schizophrenia (≥13 years old)
Schizophrenia

Bipolar 1 disorder (≥10 years old)
Bipolar 1 disorder
Lumateperone
NA
Schizophrenia


Bipolar 1 disorder
Olanzapine
Schizophrenia (≥13 years old)
Schizophrenia

Bipolar 1 disorder (≥10 years old)
Bipolar 1 disorder


Adjunctive treatment of treatment resistant depression
with fluoxetine
Paliperidone
Schizophrenia (≥12 years old)
Schizophrenia
Pimavanserin
NA
Psychosis associated with Parkinson’s disease
Quetiapine
Schizophrenia (≥13 years old)
Schizophrenia

Bipolar 1 disorder (≥10 years old)
Bipolar 1 disorder


Adjunctive treatment of treatment resistant depression
Risperidone
Schizophrenia (≥13 years old)
Schizophrenia

Bipolar 1 disorder (≥10 years old)
Bipolar 1 disorder

Irritability associated with autism spectrum disorders
(10-16 years old)

Ziprasidone
NA
Schizophrenia


Bipolar 1 disorder
1	Christian R, Saavedra L, Gaynes B. Future Research Needs for First- and Second-Generation Antipsychotics for Children and Young Adults [Internet]. In: Future Research Needs Papers. Agency for Healthcare Research and Quality (US); 2012:Appendix A, Tables of FDA-Approved Indications for First-and Second-Generation Antipsychotics. https://www.ncbi.nlm.nih.gov/books/NBK84656/
