# Supplementary material for: Systematic review: Efficacy of off-label antipsychotic use in children and adolescents with obsessive-compulsive, mood dysregulation, depression, anxiety, and sleep-related disorders
Source: Eur Child Adolesc Psychiatry. 2026 May 18;35(8):2367–81. doi: 10.1007/s00787-026-03049-9 (PMC13652945; doi:10.1007/s00787-026-03049-9)
Supplement: Supplementary file 4 [file 787_2026_3049_MOESM4_ESM.odt]

Systematic review: Efficacy of off-label antipsychotic use in children and adolescents with obsessive-compulsive, mood dysregulation, depression, anxiety, and sleep-related disorders
European Child + Adolescent Psychiatry

Authors: Akseli K. Holopainen, MS*,a; Pyry A. Vainiotalo, MS*,a; Bianca P. Arrhenius, MD, PhDb,c; Ida Ringbom, MD, PhDd; Mirja Laurila, MD, Phdd; David Gyllenberg, MD, PhDd
(*) Shared first authorship  
a University of Helsinki, Faculty of Medicine  
b Finnish Medical Society Duodecim, Helsinki, Finland 
c Department of General Practice and Primary Health Care, University of Helsinki  
d Department of Adolescent Psychiatry, Helsinki University Hospital, Helsinki, Finland

Corresponding author: David Gyllenberg. 
Email: david.gyllenberg@hus.fi
ORCID: 0000-0003-3220-9805


Online resource 2 Full search strategy including all terms and databases used in our systematic search, as well as any and all inclusion and exclusion criteria

EBM Reviews - Cochrane Central Register of Controlled Trials <October 2025> 
EBM Reviews - Cochrane Clinical Answers <October 2025> 
Ovid MEDLINE(R) ALL <1946 to October 29, 2025> 
APA PsycInfo <1806 to October 2025 Week 5>
1 (antipsych* or neurolept* or risperidone or quetiapine or clozapine or aripiprazole or lurasidone or paliperidone or ziprasidone or olanzapine or asenapine or chlorpromazine or levomepromazine or fluphenazine or loxapine or perphenazine or promazine or trifluoperazine or amisulpride or brexpiprazole or cariprazine or iloperidone or haloperidol or chlorprothixene or zuclopenthixol or flupentixol or sulpiride or sertindole or pimozide or dixyrazine or prochloroperazine or periciazine or pipotiazine or melperone or tiapride or thioridazine).mp. [mp=ti, ot, ab, fx, sh, hw, kw, tx, ct, bt, nm, kf, ox, px, rx, ui, sy, ux, mx, tc, id, tm, mf] 
2 (effect* or efficac* or efficiency or improvement).mp. [mp=ti, ot, ab, fx, sh, hw, kw, tx, ct, bt, nm, kf, ox, px, rx, ui, sy, ux, mx, tc, id, tm, mf]
3 (young* or youth* or adoles* or pediatr* or paediatr* or child* or kid or kids or underage or teen or teens or teenage*).mp. [mp=ti, ot, ab, fx, sh, hw, kw, tx, ct, bt, nm, kf, ox, px, rx, ui, sy, ux, mx, tc, id, tm, mf]
4 (clinical trial* or RCT or random* clinical trial* or random* control* trial* or random* trial* or cohort stud* or case series or uncontrol* trial* or pre-post stud*).mp. [mp=ti, ot, ab, fx, sh, hw, kw, tx, ct, bt, nm, kf, ox, px, rx, ui, sy, ux, mx, tc, id, tm, mf]
5 (depressive disorder* or anxiety disorder* or sleeping disorder* or anxiety or depression or insomnia or impulsivity or unstable or mood disorder* or mood dysregulation or mood disturbance or Disruptive mood dysregulation disorder* or Intermittent explosive disorder* or Severe mood dysregulation or MDD or DMDD or IED or SMD or EUPD or obsessive-compulsive or self-harm* or self-injury or self-mutilation or borderline or anxiety disorder* or phobi* or social anxiety or panic* or GAD or fear).mp. [mp=ti, ot, ab, fx, sh, hw, kw, tx, ct, bt, nm, kf, ox, px, rx, ui, sy, ux, mx, tc, id, tm, mf] 
6 1 and 2 and 3 and 4 and 5 
7 remove duplicates from 6
8 limit 7 to english
