# Supplementary material for: Systematic review: Efficacy of off-label antipsychotic use in children and adolescents with obsessive-compulsive, mood dysregulation, depression, anxiety, and sleep-related disorders
Source: Eur Child Adolesc Psychiatry. 2026 May 18;35(8):2367–81. doi: 10.1007/s00787-026-03049-9 (PMC13652945; doi:10.1007/s00787-026-03049-9)
Supplement: Supplementary file 5 [file 787_2026_3049_MOESM5_ESM.odt]

Systematic review: Efficacy of off-label antipsychotic use in children and adolescents with obsessive-compulsive, mood dysregulation, depression, anxiety, and sleep-related disorders
European Child + Adolescent Psychiatry

Authors: Akseli K. Holopainen, MS*,a; Pyry A. Vainiotalo, MS*,a; Bianca P. Arrhenius, MD, PhDb,c; Ida Ringbom, MD, PhDd; Mirja Laurila, MD, Phdd; David Gyllenberg, MD, PhDd
(*) Shared first authorship  
a University of Helsinki, Faculty of Medicine  
b Finnish Medical Society Duodecim, Helsinki, Finland 
c Department of General Practice and Primary Health Care, University of Helsinki  
d Department of Adolescent Psychiatry, Helsinki University Hospital, Helsinki, Finland

Corresponding author: David Gyllenberg. 
Email: david.gyllenberg@hus.fi
ORCID: 0000-0003-3220-9805


Online resource 3 List of papers excluded after full-text review with reasons of exclusion
Number
Article name
Author(s)
Reason for exclusion
1
Sequenced treatment alternatives to relieve adolescent depression (STAR-AD): a multicentre open-label randomized controlled trial protocol.
He et al.
Only research protocol available
2
Sequenced treatment alternatives to relieve adolescent depression (STAR-AD): a multicentre open-label randomized controlled trial protocol.
Stoffers-Winterling et al.
Excluded study type (review)
3
Effects of adjunctive brexpiprazole on sleep-wake and circadian parameters in youth with depressive disorders: study protocol for a clinical trial.
Carpenter et al.
Invalid population (age range 18-30 years)
4
Cognitive Outcomes with Sequential Escitalopram Monotherapy and Adjunctive Aripiprazole Treatment in Major Depressive Disorder: A Canadian Biomarker Integration Net
Chakrabarty et al.
Invalid population (age range 18-60 years)
5
Symptom Dimension of Interest-Activity Indicates Need for Aripiprazole Augmentation of Escitalopram in Major Depressive Disorder: A CAN-BIND-1 Report.
Uher et al.
Invalid population (age range 18-60 years)
6
Annual Research Review: Defining and treating pediatric treatment-resistant depression.
Dwyer et al.
Excluded study type (review)
7
Efficacy of cognitive behavioural therapy with medication for patients with obsessive-compulsive disorder: a multicentre randomised controlled trial in China
Meng et al.
Invalid intervention (SSRIs)
8
Symptomatic and Functional Outcomes and Early Prediction of Response to Escitalopram Monotherapy and Sequential Adjunctive Aripiprazole Therapy in Patients With Major Depressive Disorder: A CAN-BIND-1 Report.
Kennedy et al.
Invalid population (age range 18-60 years)
9
Aripiprazole compared with placebo for auditory verbal hallucinations in youth with borderline personality disorder: Protocol for the VERBATIM randomized controlled tri
Chanen et al.
Only research protocol available
10
A randomised, placebo-controlled 24-week study evaluating adjunctive brexpiprazole in patients with major depressive disorder.
Bauer et al.
Invalid population (age range 18-75 years)
11
Pharmacotherapy for generalized anxiety disorder in adult and pediatric patients: an evidence-based treatment review.
Strawn et al.
Excluded study type (review)
12
Effect of the Glutamate NMDA Receptor Antagonist Memantine as Adjunctive Treatment in Borderline Personality Disorder: An Exploratory, Randomised, Double-Blind, Placebo-Controlled Trial.
Kulkarni et al.
Invalid intervention (memantine)
13
Lurasidone in Children and Adolescents: Systematic Review and Case Report.
Channing et al.
Invalid population (diagnosis for psychosis in case report)
14
Risk factors and treatment for steroid-related mood and behavior symptoms in preschool children with leukemia: A case series.
Samsel et Muriel
Invalid population (no patients with our indications of choice)
15
The use, safety, and efficacy of olanzapine in a pediatric emergency department over a 10-year period
Cole et al.
Invalid population (can not extract information on patients with our indications of choice)
16
Cognitive Mediation of Symptom Change in Exposure and Response Prevention for Obsessive-Compulsive Disorder.
Su et al.
Invalid population (age range 18-65 years)
17
Efficacy of lurasidone in the treatment of major depression with mixed features: the value of early improvement as a predictor of short-term response during treatment of bipolar depression with lurasidone
Iosifescu et al.
Full text not available
18
Ziprasidone augmentation for anxious depression.
Ionescu et al.
Invalid population (age range 18-65 years)
19
Lurasidone for the treatment of major depressive disorder with mixed features: do manic symptoms moderate treatment response?
Goldberg et al.
Only poster available
20
Adjunctive brexpiprazole 1 and 3 mg for patients with major depressive disorder following inadequate response to antidepressants: a phase 3, randomized, double-blind study.
Thase et al.
Invalid population (age range 18-65 years)
21
Analysis of potentially predictive factors of efficacy of adjunct extended-release quetiapine fumarate in patients with major depressive disorder.
Bauer et al.
Invalid population (age range 18-65 years)
22
Impact on cortisol and antidepressant efficacy of quetiapine and escitalopram in depression.
Sarubin et al.
Invalid population (age range 18-65 years)
23
Efficacy of once-daily extended release quetiapine fumarate in patients with different levels of severity of generalized anxiety disorder.
Montgomery et al.
Invalid population (age range 18-65 years)
24
Depression treatment by withdrawal of short-term low-dose antipsychotic, a proof-of-concept randomized double-blind study.
Kennedy et al.
Invalid population (age range 18-65 years)
25
Pooled analysis of adjunct extended-release quetiapine fumarate in patients with major depressive disorder according to ongoing SSRI or SNRI treatment.
Bauer et al.
Invalid population (age range 18-65 years)
26
Effects of extended-release quetiapine fumarate on long-term functioning and sleep quality in patients with Generalized Anxiety Disorder (GAD): data from a randomized-withdrawal, placebo-controlled maintenance study.
Sheehan et al.
Invalid population (age range 18-65 years)
27
Augmentation of aripiprazole for depressed patients with an inadequate response to antidepressant treatment: a 6-week prospective, open-label, multicenter study.
Jon et al.
Invalid population (age range 18-65 years)
28
The effects of aripiprazole on the subscales of the Kellner Symptom Questionnaire in treatment resistant depression.
Dording et al.
Invalid population (age range 18-65 years)
29
Acute anxiolytic effects of quetiapine during virtual reality exposure--a double-blind placebo-controlled trial in patients with specific phobia.
Diemer et al.
Invalid population (age range 18-70 years)
30
Extended-release quetiapine fumarate (quetiapine XR) monotherapy and quetiapine XR or lithium as add-on to antidepressants in patients with treatment-resistant major depressive disorder.
Bauer et al.
Invalid population (age range 18-65 years)
31
Efficacy of extended release quetiapine fumarate monotherapy in patients with major depressive disorder: a pooled analysis of two 6-week, double-blind, placebo-controlled studies.
Weisler et al.
Invalid population (age range 18-65 years)
32
Long-term functioning and sleep quality in patients with major depressive disorder treated with extended-release quetiapine fumarate.
Sheehan et al.
Invalid population (age range 18-65 years)
33
A double-blind, placebo-controlled study of aripiprazole adjunctive to antidepressant therapy among depressed outpatients with inadequate response to prior antidepressant therapy (ADAPT-A Study).
Fava et al.
Invalid population (age range 18-65 years)
34
Aripiprazole augmentation strategy in clomipramine-resistant depressive patients: an open preliminary study.
Fabrazzo et al.
Invalid population (age range 18-65 years)
35
The effectiveness and safety of adjunctive aripiprazole in Taiwanese patients with antidepressant-refractory major depressive disorder: a prospective, open-label trial.
Chen et al.
Invalid population (age range 25-50 years)
36
The comparison of aripiprazole and risperidone augmentation in selective serotonin reuptake inhibitor-refractory obsessive-compulsive disorder: a single-blind, randomised study.
Selvi et al.
Invalid population (age range 18-65 years)
37
A case series of aripiprazole augmentation of selective serotonin reuptake inhibitors in treatment-refractory obsessive compulsive disorder.
Matsunaga et al.
Invalid population (age range 18-65 years + developmental disability)
38
Effectiveness and tolerability of aripiprazole in a real-world outpatient population of youth.
Bildik et al.
Invalid population (can not extract information on patients with our indications of choice)
39
A double-blind, randomized, controlled trial of fluoxetine plus quetiapine or clomipramine versus fluoxetine plus placebo for obsessive-compulsive disorder.
Diniz et al.
Invalid population (age range 18-65 years)
40
Efficacy and tolerability of extended release quetiapine fumarate (quetiapine XR) monotherapy in major depressive disorder: a placebo-controlled, randomized study.
Bortnick et al.
Invalid population (age range 18-65 years)
41
Olanzapine/fluoxetine combination in patients with treatment-resistant depression: rapid onset of therapeutic response and its predictive value for subsequent overall response in a pooled analysis of 5 studies.
Tohen et al.
Excluded study type (review)
42
A pooled MADRS/IDS cross-correlation analysis: clinician and patient self-report assessment of improvement in core depressive symptoms with adjunctive aripiprazole.
Reimherr et al.
Invalid population (age range 18-65 years)
43
Quetiapine: novel uses in the treatment of depressive and anxiety disorders.
Ravindran et al.
Excluded study type (review)
44
Efficacy and tolerability of extended release quetiapine fumarate monotherapy as maintenance treatment of major depressive disorder: a randomized, placebo-controlled trial.
Liebowitz et al.
Invalid population (age range 18-65 years)
45
Long-term efficacy and tolerability of clozapine combined with ziprasidone or risperidone.
Kuwilsky et al.
Invalid population (patients had schizophrenia) Invalid population (patients had schizophrenia)
46
Quetiapine versus clomipramine in the augmentation of selective serotonin reuptake inhibitors for the treatment of obsessive-compulsive disorder: a randomized, open-label trial.
Diniz et al.
Invalid population (age range 18-65 years)
47
A pooled analysis of two randomised, placebo-controlled studies of extended release quetiapine fumarate adjunctive to antidepressant therapy in patients with major depressive disorder.
Bauer et al.
Invalid population (age range 18-65 years)
48
Extended-release quetiapine fumarate (quetiapine XR): a once-daily monotherapy effective in generalized anxiety disorder. Data from a randomized, double-blind, placebo- and active-controlled study.
Bandelow et al.
Invalid population (age range 18-65 years)
49
Quetiapine augments the effect of citalopram in non-refractory obsessive-compulsive disorder: a randomized, double-blind, placebo-controlled study of 76 patients.
Vulink et al.
Invalid population (ages over 18 years)
50
Aripiprazole augmentation in major depressive disorder: a double-blind, placebo-controlled study in patients with inadequate response to antidepressants.
Berman et al.
Invalid population (age range 18-65 years)
51
Quetiapine augmentation in depressed patients with partial response to antidepressants.
Olver et al.
Invalid population (age range 18-60 years)
52
Olanzapine plus dialectical behavior therapy for women with high irritability who meet criteria for borderline personality disorder: a double-blind, placebo-controlled pilot study.
Linehan et al.
Invalid population (age range 18-60 years)
53
Aripiprazole as an adjunctive treatment for refractory unipolar depression.
Hellerstein et al.
Invalid population (age range 18-70 years)
54
Predictors of aripiprazole treatment continuation in hospitalized patients.
Coley et al.
Invalid outcome (did not study efficacy)
55
Quetiapine as monotherapy for social anxiety disorder: a placebo-controlled study.
Vaishnavi et al.
Invalid population (age range 18-65 years)
56
A randomized, double-blind comparison of olanzapine/fluoxetine combination, olanzapine, and fluoxetine in treatment-resistant major depressive disorder.
Thase et al.
Invalid population (age range 18-65 years)
57
Quetiapine addition in obsessive-compulsive disorder: is treatment outcome affected by type and dose of serotonin reuptake inhibitors?.
Denys et al.
Invalid population (age range 18-65 years)
58
A double-blind, randomised, controlled clinical trial of acetyl-L-carnitine vs. amisulpride in the treatment of dysthymia.
Zanardi et Smeraldi
Invalid population (age range 18-60 years)
59
Effects of risperidone augmentation in patients with treatment-resistant depression: Results of open-label treatment followed by double-blind continuation.
Rapaport et al.
Invalid population (age range 18-85 years)
60
Efficacy of pharmacotherapy against core traits of borderline personality disorder: meta-analysis of randomized controlled trials.
Nose et al.
Excluded study type (meta-analysis)
61
Pharmacotherapies in the management of obsessive-compulsive disorder.
Blier et al.
Excluded study type (review)
62
Risperidone for the Treatment of Affective Symptoms in Children with Disruptive Behavior Disorder: A Post Hoc Analysis of Data from a 6-Week, Multicenter, Randomized, Double-Blind, Parallel-Arm Study.
Biederman et al.
Invalid study population (no patients with indications of choice)
63
Open-label study of atypical neuroleptic quetiapine for treatment of borderline personality disorder: impulsivity as main target.
Villeneuve et Lemelin
Invalid population (age range 18-60 years)
64
A preliminary case series on the use of quetiapine for posttraumatic stress disorder in juveniles within a youth detention center.
Stathis et al.
Invalid population (patients had PTSD)
65
Low-dose risperidone augmentation of fluvoxamine treatment in obsessive-compulsive disorder: a double-blind, placebo-controlled study.
Erzegovesi et al.
Invalid population (age range 18-65 years)
66
Quetiapine augmentation of SRIs in treatment refractory obsessive-compulsive disorder: a double-blind, randomised, placebo-controlled study [ISRCTN83050762].
Carey et al.
Invalid population (age range 18-65 years)
67
A preliminary, randomized trial of fluoxetine, olanzapine, and the olanzapine-fluoxetine combination in women with borderline personality disorder.
Zanarini et al.
Invalid population (age range 18-40 years)
68
Psychopharmacological treatment of depression, anxiety, irritability and insomnia in patients receiving interferon-alpha: a prospective case series and a discussion of biological mechanisms.
Maddock et al.
Invalid population (age range 18-61 years)
69
Patterns of concomitant psychotropic medication use during a 2-year study comparing clozapine and olanzapine for the prevention of suicidal behavior.
Glick et al.
Invalid population (age range 18-65 years)
70
Olanzapine versus placebo in the treatment of borderline personality disorder.
Bogenschutz et al.
Invalid population (age range 18-60 years)
71
The effectiveness of risperidone in the treatment of three children with feeding disorders.
Berger-Gross et al.
Invalid population (patients had feeding disorders)
72
Treatment of previously undiagnosed psychiatric disorders in persons with developmental disabilities decreased or eliminated self-injurious behavior.
Tsiouris et al.
Invalid population (patients had intellectual disability)
73
Quetiapine augmentation of serotonin reuptake inhibitors in obsessive-compulsive disorder.
Mohr et al.
Invalid population (age range 19-61 years)
74
An open pilot study combining risperidone and a selective serotonin reuptake inhibitor as initial antidepressant therapy.
Hirose et al.
Invalid population (age range 18-81 years)
75
Open trial study of a combined antidepressant (amitryptiline, perphenazine, diazepam) versus fluoxetine or imipramine in ambulatory depressed patients.
Diaz et al.
Full article can not be found
76
Efficacy of olanzapine in social anxiety disorder: a pilot study.
Barnett et al.
Invalid population (age range 18-65 years)
77
Olanzapine treatment of female borderline personality disorder patients: a double-blind, placebo-controlled pilot study.
Zanarini et al.
Invalid population (age range 18-40 years)
78
Risperidone treatment of children and adolescents with pervasive developmental disorders: a prospective open-label study.
McDougle et al.
Invalid population (patients had intellectual disability)
79
Use of risperidone in pervasive developmental disorders: a case series.
Fisman et al.
Invalid population (patients had pervasive development disorder)
80
Haloperidol addition in fluvoxamine-refractory obsessive-compulsive disorder. A double-blind, placebo-controlled study in patients with and without tics.
McDougle et al.
Invalid population (age range 18-65 years)
81
Psychopharmacology of disorders in children.
Sylvester et al.
Excluded study type (review)
82
Bromazepam in generalized anxiety. Randomized, multi-practice comparisons with both chlorprothixene and placebo.
Kragh-Sorensen et al.
Invalid population (age range 18-60 years)
83
Progress in pharmacotherapy of borderline disorders. A double-blind study of amitriptyline, haloperidol, and placebo.
Soloff et al.
Invalid population (results for under 18-year-olds not extractable)
84
Separate and combined anxiolytic and anti-depressant treatment of mixed anxiety/depression. A double-blind, placebo controlled comparison. Sussex Clinical Trials Group.
Brodie et al.
Invalid population (ages 18 and over)
85
Melperone in low doses in anxious neurotic patients. A double-blind placebo-controlled clinical study.
Poldinger et al.
Invalid population (age range 18-60 years)
86
Treatment of depressive anxiety states associated with psychosomatic symptoms. A double-blind multicentre clinical study: mianserin versus melitracen-flupentixol.
Van Moffaert et al.
Full article can not be found
87
Mixed anxiety/depressive illness in general practice. A therapeutic comparison of nomifensine with fluphenazine/nortriptyline.
Valle-Jones et al.
Invalid population (age 18 and over)
88
Response of borderline patients to loxapine and chlorpromazine.
Leone et al.
Full article can not be found
89
A double-blind comparison of flupenthixol, nortriptyline and diazepam in neurotic depression.
Johnson et al.
Invalid population (results for under 18-year-olds not extractable)
90
Clinical use of haloperidol (Serenace) in child psychiatry
Nagaraja et al.
Full article can not be found
91
Once-daily treatment for mixed anxiety/depressive states: a comparison of slow release amitriptyline and fluphenazine with nortriptyline.
Magnus et al.
Full article can not be found
92
Effect of flupenthixol on depression with special reference to combination use with tricyclic antidepressants.
Fujiwara et al.
Invalid population (results for under 18-year-olds not extractable)
93
Once daily administration of a fluphenazine/nortriptyline preparation in the treatment of mixed anxiety/depressive states.
Brodie et al.
Invalid population (age range 18-65 years)
94
A comparison of amitriptyline and a fluphenazine/nortriptyline preparation in anxiety-depressive states.
Bennie et al.
Full article can not be found
95
Sulpiride vs. amitriptyline in the treatment of depression.
Niskanen et al.
Full article can not be found
96
A combination of anti-anxiety drugs: a report from the General Practitioner Research Group.
No authors listed
Full article can not be found
97
A guide for drug use in depressive disorders.
Raskin et al.
Excluded study type (review)
98
A controlled outpatient trial of perphenazine-amitriptyline and chlorpromazine.
Haskell et al.
Invalid population (age range 18-45 years)
99
Haloperidol versus diazepam: a double-blind crossover clinical trial
Lord et al.
Invalid population (results for under 18-year-olds not extractable)
100
A comparative study of doxepin versus trifluoperazine in anxiety neurosis.
Kishore et al.
Full article can not be found
101
The computer analyzes two psychotherapeutic agents in anxiety/depressed states.
Falk et al.
Full article can not be found
102
Perphenazine-amitriptyline in neurotic underachieving students: A controlled study.
Weise et al.
Full article can not be found
103
Thioridazine as an antidepressant.
No authors listed
Full article can not be found
104
Pimozide in anxiety neurosis.
No authors listed
Full article can not be found
105
Anxious-depressed adults and problem children treated with thioridazine in private practice.
Scweikart et al.
Full article can not be found
106
Antidepressant effects of tranquillizers.
No authors listed
Full article can not be found
107
Psychotropics in anxiety and depression: Combination or single agent?
Ucko et al.
Full article can not be found
108
Differential response to chlorpromazine, imipramine, and placebo: A study of subgroups of hospitalized depressed patients.
Raskin et al.
Full article can not be found
109
Comparative effects of fluphenazine, fluphenazine-chlordiazepoxide and fluphenazine-imipramine.
Hanlon et al.
Full article can not be found
110
Feasibility of evaluating psychopharmaceutical agents in cooperative studies in private practice.
Free et al.
Full article can not be found
111
Controlled trials of an amitriptyline-fluphenazine combination in depressive neuroses and psychoses: a collaborative study.
Baldini et al.
Full article can not be found
112
Depression: Prognosis and drug treatment.
Rose et al.
Full article can not be found
113
Psychoactive drugs in the treatment of emotionally disturbed children: Haloperidol and diazepam.
Lucas et al.
Full article can not be found
114
Biphasic drugs in the treatment of depression with anxiety.
Krakowski et al.
Full article can not be found
115
High dosage chlorpromazine alone and in combination with an antiparkinsonian agent (procyclidine) in the treatment of hospitalized depressions.
Raskin et al.
Invalid population (results for under 18-year-olds not extractable)
116
Psychiatric diagnosis and a typology of clinical drug effects.
Klein et al.
Invalid population (patient age not specified)
117
Importance of psychiatric diagnosis in prediction of clinical drug effects.
Klein et al.
Full article can not be found
118
A clinical study of the anxiolytic effect of dixyrazine (Esucos).
Hakola et al.
Full article can not be found
119
The role of personality variables in response to chlorpromazine, dextroamphetamine and placebo.
Forrest et al.
Invalid population (age range 18-30 years)
120
The effect of a protriptyline-perphenazine combination in chronic psychiatric patients.
St-Jean et al.
Full article can not be found
121
Combined amitriptyline-perphenazine (Etrafon) compared with chlordiazepoxide hydrochloride: a double-blind, crossover study.
Martins et al.
Full article can not be found
122
High-intensity transcranial alternating current stimulation combined with pharmacotherapy for adolescent major depressive disorder: a prospective case report study
Yuan et al.
Did not study efficacy of antipsychotics
123
Transdiagnostic Efficacy of Cariprazine: A Systematic Review and Meta-Analysis of Efficacy Across Ten Symptom Domains
Barabassy et al.
Invalid population (only adult studies included)
124
Lumateperone as Adjunctive Therapy in Patients With Major Depressive Disorder: Results From a Randomized, Double-Blind, Phase 3 Trial
Durgam et al.
Invalid population (age range 18-65 years)
125
Real-world effectiveness of cariprazine in major depressive disorder and bipolar I disorder in the United States
McIntyre et al.
Invalid population (only 18+ year-olds included)
126
A randomised study and an extension study of brexpiprazole in patients with borderline personality disorder
Rothman et al.
Invalid population (only adult patients included)
127
Efficacy of esketamine nasal spray over quetiapine extended release over the short and long term: sensitivity analyses of ESCAPE-TRD, a randomised phase IIIb clinical trial
Young et al.
Invalid population (age range 18-74 years)
128
Case Report: Functional and Symptomatic Improvement With Cariprazine in Various Psychiatric Patients: A Case Series
Vannucchi et al.
Invalid population (only adult patients included) and study indications (psychosis and bipolar disorder)
129
Intramuscular ketamine vs. escitalopram and aripiprazole in acute and maintenance treatment of patients with treatment-resistant depression: A randomized double-blind clinical trial
Cigognini et al.
Invalid population (age range 18-40 years)
130
Clinical utility of trifluoperazine in the treatment of obsessive-compulsive disorder: A case report

Chithra et al.
Invalid population (patient was 19 years-old)
131
Treatment With Quetiapine for Youth With Substance Use Disorders and Severe Mood Dysregulation
Yule et al.
Full article can not be found

132
Methylphenidate vs. Risperidone for the Treatment of Children and Adolescents With ADHD and Disruptive Disorders
Gothelf et al.
Invalid population (oppositional defiant disorder, attention-deficit hyperactivity disorder, conduct disorder)
